# A myofiber-derived secreted factor for muscle regeneration

**DOI:** 10.1093/lifemeta/load025

**Published:** 2023-06-14

**Authors:** Fang Xiao, Zheng Sun

**Affiliations:** Department of Cadress Medical Care and Geriatrics, The Second Hospital, Cheeloo College of Medicine, Shandong University, Jinan, Shandong 250033, China; Department of Medicine—Division of Endocrinology, Diabetes, and Metabolism, Baylor College of Medicine, Houston, TX 77030, United States; Department of Molecular and Cellular Biology, Baylor College of Medicine, Houston, TX 77030, United States

**A recent study shed light on transcriptional regulation of myofiber-derived Dkk3, a secreted protein involved in muscle differentiation, which has therapeutic implications in damage-induced muscle regeneration and obesity-associated muscle atrophy.**

Studies in muscle regeneration have been centered on resident muscle stem cells (MuSCs), also known as satellite cells. Upon muscle damage, quiescent MuSCs are activated to enter the cell cycle and start proliferation. They give rise to myogenic progenitor cells or myoblasts that differentiate into myocytes. Myocytes fuse with each other to form multinucleate myotubes that further mature to become myofibers [1]. Each of the above stages is characterized by cascades of gene expression patterns orchestrated by distinct transcription factors receiving external signals from the microenvironment in alliance with physical interactions between the differentiating muscle cells and the other cell types in the muscle [2].

Xu *et al*. found a surprising phenomenon during their initial study of the muscle-regenerating role of their favorite gene *Baf60c* [3]. Myofiber-specific knockout of *Baf60c* (MKO) using MLC-Cre in mice does not cause abnormal differential defects at the baseline but a robust defect in muscle regeneration capacity after drug-induced muscle damage. By comparison, the MuSC-specific knockout of *Baf60c* using Pax7-CreER causes a much milder effect. To explore the potential myofiber-MuSC communications, the investigators established an *in vitro* assay by obtaining conditioned medium (CM) from cultured C2C12 myotubes and then adding the CM onto primary single myofibers isolated from adult mice to observe the effects of CM on myotube differentiation and maturation. CM from C2C12 myotubes treated with siRNA targeting *Baf60c* is less competent than control siRNA (shCTR) in supporting primary myotube differentiation (Table 1), suggesting that a myofiber-derived secreted factor contributes to defective muscle regeneration.

**Table 1:** Summary of the key interventional experiments.

*In vivo*			*In vitro*	
Manipulation in mice	Baseline regeneration	CTX-induced regeneration	Manipulation in C2C12	C2C12-CM on differentiation
Baf60c-MKO vs. WT	↔	↓	siBaf60c	↓
AAV-Dkk3 vs. GFP	↔	↓	Dkk3-Fc	↓
AAV-shDkk3 vs. shCTR	↔	↔		
AAV-shDkk3 vs. shCTR, in MKO		↑		
MCK-Baf60c transgenic (TG)		↑	Baf60c OE	↑
AAV-Dkk3 vs. GFP, in TG		↓		
AAV-shDkk3 vs. shCTR, in ob/HFD		↑		

CTX, cardiotoxin; CM, conditioned medium; CTR, control; OE, overexpression.

↔ No major change.

↓ Impaired muscle regeneration or differentiation.

↑ Improved muscle regeneration or differentiation.

What is the secreted factor? Since Baf60c is a component of the SWI/SNF chromatin-remodeling complex with the known main function in gene transcription, the investigators focused on transcripts for secreted factors in their microarray analysis. They identified that Dkk3 was upregulated by 5- to 10-fold upon *Baf60c* knockout at both RNA and protein levels. Dkk3 is a secreted factor previously shown to inhibit smooth muscle differentiation [4, 5] and muscle atrophy [6]. The investigators then performed rigorous and comprehensive experiments to characterize the role and interrelationship between Baf60c and Dkk3 in muscle regeneration.

AAV-mediated overexpression of *Dkk3* mimicked *Baf60c* MKO in impairing muscle regeneration after drug-induced damage in mice, while adding purified Dkk3 to the differentiation medium in the isolated primary single myofibers also impaired myotube differentiation (Table 1). Conversely, AAV-mediated shRNA targeting *Dkk3* did not cause an apparent change in wild-type mice but rescued the defective muscle regeneration in the *Baf60c* MKO mice (Table 1). These results demonstrate that Dkk3 upregulation is sufficient and required for defective muscle regeneration in *Baf60c* MKO mice. Mechanistically, Baf60c co-localizes with a transcription factor Six4 on the promoter of *Dkk3* and likely serves as a corepressor to suppress *Dkk3* transcription.

Could the Baf60c-Dkk3 pathway be harnessed to improve muscle regeneration? Transgenic overexpression of *Baf60c* using MCK promoter enhanced muscle regeneration after drug-induced muscle damage, an effect abolished by AAV-mediated overexpression of *Dkk3* (Table 1). Muscle *Baf60c* gene expression negatively correlates with obesity from the investigators’ previous study [7, 8], in line with their new data demonstrating a positive correlation between *Dkk3* RNA and protein levels with obesity in human muscle and plasma samples as well as muscle samples from mouse models. AAV-shRNA knockdown of *Dkk3* improved obesity-associated muscle regeneration defect in mice, suggesting that it is a potential drug target for treating obesity-associated muscle weakness.

These comprehensive results from rigorous experimental design demonstrate translational values of targeting Baf60c-Dkk3 in muscle regeneration. They also raise interesting questions that warrant further investigations. (i) What is the identity of the target cells that Dkk3 acts on? If it is MuSC, at what stages (activation, proliferation, differentiation, fusion, or maturation) does Dkk3 act? (ii) What are the Dkk3 receptor(s) and downstream molecular changes in its target cell? Are the receptor(s) or the downstream molecular changes in MuSC required for the Dkk3-mediated effects in muscle regeneration? (iii) What is the cause of the obesity-associated changes in *Baf60c* expression? Are other physiological or pathological conditions associated with Baf60c loss-of-function independent of its RNA or protein levels? (iv) Mild obesity can be associated with increased muscle mass in young human populations [9, 10]. What is the role of Baf60c and Dkk3 in this scenario? How about aging-associated muscle atrophy or cancer-related sarcopenia? (v) How can Baf60c or Dkk3 be therapeutically targeted in humans?

## References

[1] Schmidt M, Schüler SC, Hüttner SS et al. Cell Mol Life Sci 2019;76:2559–70. 30976839 10.1007/s00018-019-03093-6 PMC6586695

[2] Relaix F, Bencze M, Borok MJ et al. Nat Commun 2021;12:692. 33514709 10.1038/s41467-020-20760-6 PMC7846784

[3] Xu J, Li X, Chen W et al. J Exp Med 2023;220:e20221123.37284884

[4] Karamariti E, Margariti A, Winkler B et al. Circ Res 2013;112:1433–43. 23529184 10.1161/CIRCRESAHA.111.300415

[5] Wang X, Karamariti E, Simpson R et al. J Biol Chem 2015;290:19844–52. 26105053 10.1074/jbc.M115.641415 PMC4528144

[6] Yin J, Yang L, Xie Y et al. Nat Commun 2018;9:1752. 29717119 10.1038/s41467-018-04038-6 PMC5931527

[7] Meng ZX, Li S, Wang L et al. Nat Med 2013;19:640–5. 23563706 10.1038/nm.3144 PMC3650110

[8] Meng ZX, Wang L, Xiao Y et al. Diabetes 2014;63:1533–45. 24458360 10.2337/db13-1061 PMC3994956

[9] Ten Hoor GA, Plasqui G, Schols AMWJ et al. Sports Med Open 2018;4:12. 29492711 10.1186/s40798-018-0125-4 PMC5833324

[10] Tomlinson DJ, Erskine RM, Morse CI et al. Biogerontology 2016;17:467–83. 26667010 10.1007/s10522-015-9626-4 PMC4889641

